# The Gut Microbiota and Its Metabolites and Their Association with the Risk of Autoimmune Thyroid Disease: A Mendelian Randomization Study

**DOI:** 10.3390/nu16223898

**Published:** 2024-11-15

**Authors:** Chenyu Zhang, Weiping Teng, Chuyuan Wang, Zhongyan Shan

**Affiliations:** Department of Endocrinology and Metabolism, The First Affiliated Hospital of China Medical University, Institute of Endocrinology, NHC Key Laboratory of Diagnosis and Treatment of Thyroid Diseases, China Medical University, Shenyang 110001, China; zhangchenyu@cmu.edu.cn (C.Z.); twp@vip.163.com (W.T.)

**Keywords:** gut microbiota, metabolites, autoimmune thyroid disease, 3-indoleglyoxylic acid, mendelian randomization study

## Abstract

**Objectives**: Observational research shows associations of the gut microbiota and its metabolites with autoimmune thyroid disease (AITD), but the causality is undetermined. **Methods**: Two-sample Mendelian randomization (MR) was employed to analyze the association of the gut microbiota and its metabolites with AITD. A total of 119 gut microbiotas and nine fecal/circulating metabolites were the exposures. AITD, Graves’ disease (GD), and Hashimoto’s thyroiditis (HT) were the outcomes. Inverse-variance weighting (IVW) was primarily used to assess causality; Cochran’s Q was used to assess heterogeneity. Sensitivity analyses (weighted median, MRPRESSO regression, MRPRESSO intercept, MRPRESSO global, Steiger filtering, leave-one-out) were conducted to assess causal estimate robustness. Multivariable MR (MVMR) was used to estimate the effects of body mass index (BMI) and alcohol consumption frequency on causality. **Results**: The outcomes were potentially causally associated with 22 gut microbiotas and three metabolites. After multiple-test correction, 3-indoleglyoxylic acid retained significant causality with AITD (IVW: odds ratio [OR] = 1.09, 95% confidence interval [CI] = 1.05–1.14, *p* = 2.43 × 10^−5^, FDR = 0.009). The sensitivity analyses were confirmatory (weighted median: OR = 1.06, 95% CI = 1.01–1.12, *p* = 0.025; MRPRESSO: OR = 1.09, 95% CI = 1.15–1.14, *p* = 0.001). MVMR revealed no confounding effects on this association (BMI: OR = 1.21, 95% CI =1.08–1.35, *p* = 0.001; drinks/week: OR = 1.22, 95% CI = 1.04–1.43, *p* = 0.014). **Conclusions**: MR revealed no significant causal effects of the gut microbiota on the outcomes. However, MR revealed the causal effects of 3-indoleglyoxylic acid on the risk of AITD.

## 1. Introduction

Autoimmune thyroid disease (AITD) is the most prevalent autoimmune disease and the most common thyroid disorder [1]. AITD can result in thyroid dysfunction, cardiovascular disease [2], autoimmune encephalitis [3], and adverse pregnancy outcomes [4]. The two extremes of AITD are Hashimoto’s thyroiditis (HT) and Graves’ disease (GD) [5]. In addition to genetic factors, environmental factors (radiation, viral infections, medications, selenium, and iodine) can influence the incidence of AITD [5,6,7]. Recent studies have identified changes in the gut microbial composition as another potential environmental risk factor for AITD [8].

The gut microbiota plays a crucial role in the immune response. Certain microbiota species continuously present antigens that dictate the host’s immune response [9], fostering the differentiation and maturation of regulatory T cells [10] and modulating the immune reactivity of B cells, dendritic cells, and macrophages [9]. Gut microbes produce metabolites that may affect the immune system via the bloodstream [11], including short-chain fatty acids (SCFAs) [12], trimethylamine *N*-oxide (TMAO) [13], tryptophan derivatives, and bile acids (BAs) [14]. Additionally, lipopolysaccharides (LPSs), which are endotoxins produced by pathogenic bacteria, can potentially induce intestinal inflammation, compromise intestinal barrier integrity, and influence immune function [15].

Numerous studies have reported a link between the gut microbiota and AITD. A meta-analysis concluded that, compared with those in the control group, the abundances of *Bifidobacterium* and *Lactobacillus* in AITD were significantly lower, while the abundance of *Bacteroides fragilis* was significantly greater [16]. Two studies reported a decreased abundance of *Prevotella* in HT patients [17,18]. For GD patients, the abundances of *Lactobacillus* were correlated with thyroid-stimulating hormone receptor antibodies (TRAb), thyroid peroxidase antibodies (TPOAb), and thyroglobulin antibodies (TgAb) [19,20]; *Synergistetes* showed a negative association with thyroid-stimulating antibody (TSAb); and *Ruminococcus*, *Bifidobacterium*, and *Veillonella* were positively correlated with TRAb [21,22]. Other bacterial groups such as *Prevotella 9*, *Actinomyces odontolyticus*, and *Negativicutes* are positively associated with TPOAb [23]. Furthermore, the gut microbiota significantly impacts the intestinal absorption of iodine and selenium [24,25,26]. For example, *Bacteroides* can convert intracellular selenite into selenocysteine and selenomethionine, potentially enhancing organic selenium absorption [27] and contributing to a reduction in thyroid autoantibodies.

Metabolites also play a critical role in AITD development. SCFAs, through their interaction with G-protein-coupled receptors (GPCRs) on leukocytes and intestinal epithelial cells [28], can inhibit AITD. SCFA supplementation has been shown to upregulate IL-22 and IL-10, suppress AITDs and the NF-κB inflammatory pathway, reduce LPS stimulation, and regulate TNF-α expression [29]. In individuals with primary hypothyroidism, primarily due to AITD, a decrease in SCFA-producing bacteria leads to decreased SCFA levels, exacerbating damage to the intestinal barrier and increasing serum LPS levels [30]. Additionally, SCFAs, in conjunction with the sodium/iodide symporter, may enhance iodide uptake by thyroid follicular cells [31]. Gut microbiota metabolites also hold diagnostic potential in other thyroid disorders. In a mouse model of Graves’ ophthalmopathy, fecal 16S rRNA functional predictions revealed significant changes in BA metabolism [32]; TMAO has shown potential in distinguishing between benign and malignant thyroid nodules [33]; and indole-3-carboxaldehyde in blood is a biomarker for papillary thyroid cancer [34]. While numerous studies have been conducted, their findings are often inconsistent, likely due to differences in detection methods, diet, and lifestyle, which contribute to substantial interindividual heterogeneity. Additionally, most studies have small sample sizes, limiting their ability to establish a strong relationship between the gut microbiota and AITD. Finally, as case–control studies, they are limited in assessing causality between the gut microbiota and AITD, adding to the challenge of fully understanding this relationship.

Randomized controlled studies focused on the gut microbiota may contribute to establishing causal relationships; however, high-quality evidence is lacking in the field of the gut–thyroid axis. In such scenarios, Mendelian randomization (MR) can serve as an alternative to randomized controlled trials, facilitating the exploration of causality [35]. The key idea of MR is to utilize the random allocation of genetic variation in nature, treating genetic variation as an instrumental variable (IV), effectively minimizing potential confounding factors like environmental and socioeconomic influences to enable more reliable causal assessment [35]. MR studies often leverage large genome-wide association study (GWAS) datasets, increasing their power to detect small effect sizes and enhancing their statistical robustness. By using genetic variation as an IV, MR also ensures that exposure (e.g., gut microbiota traits) precedes the outcome (AITD), effectively mitigating reverse causation bias. Therefore, MR is well suited for causal inference in studies examining the relationship between the gut microbiota and disease.

In this study, we used statistics from several GWASs of gut microbes and fecal/circulating metabolites to assess potential causal associations of gut microbes and metabolites with AITD using two-sample MR, and multivariate MR (MVMR) was used to adjust for possible confounders (body mass index [BMI] and alcohol consumption). Based on these analyses, we investigated the role of the gut microbiome in the pathogenesis of AITD, aiming to provide evidence for the emergence of innovative therapeutic strategies, including dietary interventions, probiotic supplementation, and fecal microbiota transplantation.

## 2. Materials and Methods

### 2.1. Study Design and Data Sources

**Figure 1:**
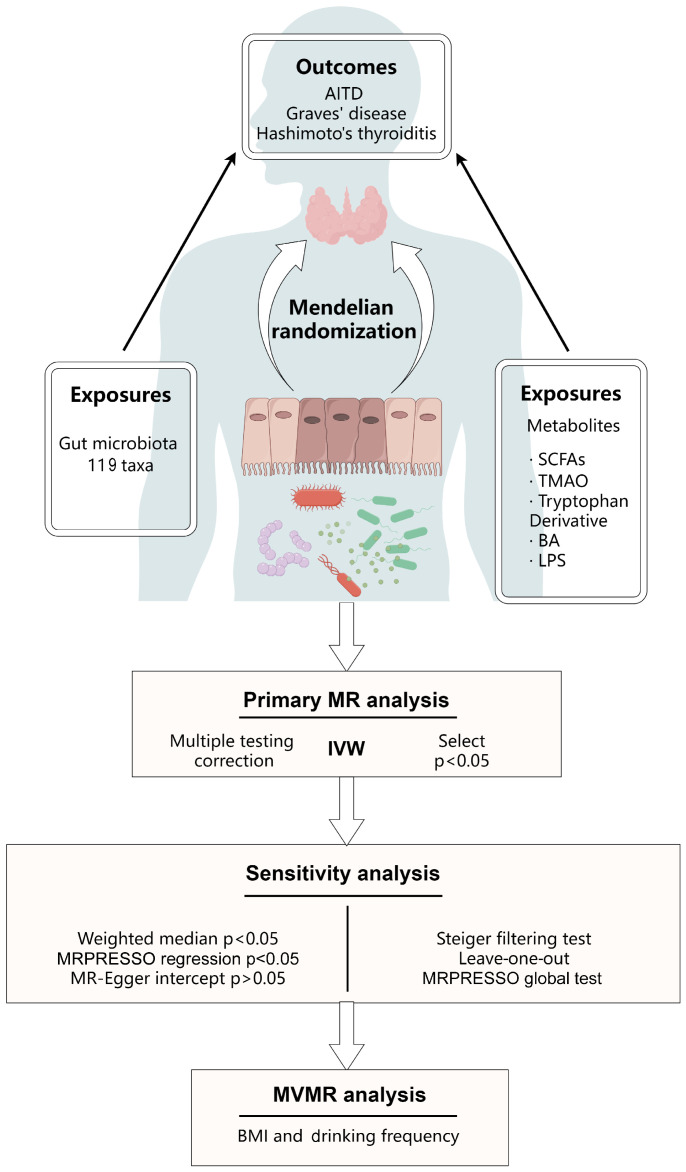
An overview of the Mendelian randomization design used to investigate the causal effect. AITD, autoimmune thyroid disease; BA, bile acid; IVW, inverse-variance weighting; LPS, lipopolysaccharide; MR, mendelian randomization; MRPRESSO, Mendelian randomization pleiotropy residual sum and outlier; MVMR, multivariate mendelian randomization; SCFAs, short-chain fatty acids; TMAO, trimethylamine *N*-oxide.

This study’s results are reported in accordance with the STROBE-MR guidelines (Supplementary Table S1) [36]. Furthermore, such an MR study must satisfy three assumptions for the instrumental variables (IVs) [37]: (A) it predicts the exposure of interest; (B) it is independent of potential confounders; and (C) it influences the outcome only through risk factors. Figure 1 depicts the research design and assumptions for the MR analysis. Initially, we conducted a two-sample MR analysis of 119 different gut microbiotas and AITD. Then, based on reports in the literature (Supplementary Table S2), we identified nine gut microbiota metabolites in five categories that may be related to AITD. These SCFAs include three SCFAs (fecal butyrate, circulating acetate, and propionate) [38,39], circulating TMAO [40], two BAs (circulating cholic acid and taurocholic acid) [32], two indoles and their derivatives (circulating indole-3-propionate and 3-indoleglyoxylic acid) [41,42], and LPS (proxied by 3-hydroxymyristate) [43,44].

Supplementary Table S3 provides detailed information on the data sources. The first part of the exposure data comprises gut microbiota GWAS data from the MiBioGen consortium (https://mibiogen.gcc.rug.nl/, accessed on 7 June 2023) [45]. This dataset included genome-wide genotype data and 16S rRNA fecal microbiome profiles obtained from 18,340 individuals across 24 cohorts. We included only individuals of European descent (n = 13,266) in our study. The taxonomic resolution of the fecal microbiome in this research extended to the genus level. A total of 119 bacterial genera were extracted. We used independent SNPs (r^2^ ≤ 0.01) as genetic tools and selected 116 genera (*p* < 1 × 10^−5^) for subsequent analyses, excluding those with fewer than three SNPs.

The second part of the GWAS exposure data on fecal and blood metabolites came from five different studies (Supplementary Table S3). The IVs for the fecal butyric acid metabolic pathway (PWY-5022) originated from the GWAS conducted in the Dutch Microbiome Project by Lopera-Maya EA et al. [46]. The IVs for circulating acetic acid were derived from a pooled GWAS comprising 14 European cohorts encompassing 24,925 individuals, as reported by Kettunen J et al. [47]. IVs associated with circulating propionate and TMAO were extracted from a cohort of 822 participants in the Chronic Renal Insufficiency Cohort (CRIC) study conducted by Rhee EP et al. [48]. IVs concerning indole and its derivatives (circulating indole-3-propionate and 3-indole glyoxylic acid) were acquired from GWASs on blood metabolomics conducted within the KORA and TwinsUK European cohorts by Shin et al. [49] and the Canadian Longitudinal Study on Aging (CLSA) cohort by Chen Y et al. [50], respectively. Circulating cholic acid, taurocholic acid, and LPS (proxied by 3-hydroxymyristate [44]) IVs were also obtained from the study by Chen Y et al. [50]. The selection criteria for all IVs were r^2^ ≤ 0.01 and *p* < 1 × 10^−5^.

There were three genetic outcome-traits in this study. GWAS summary statistics for AITD were acquired from the Icelandic and UK biobanks (https://www.ukbiobank.ac.uk/, accessed on 7 June 2023) [6], encompassing 755,406 individuals (30,234 patients and 725,172 control participants) of European descent. The phenotype ‘AITD’ was specifically characterized as encompassing GD, HT, other forms of hypothyroidism, and/or treatment with thyroxine, excluding known non-AITD and drug-induced thyroid conditions [6]. The GWAS summary data for GD and HT were obtained from a recent large-scale meta-analysis of 220 phenotypes [51], which drew on data from three prominent biobanks: BioBank Japan (https://biobankjp.org/en/, accessed on 7 June 2023), UK Biobank (https://www.ukbiobank.ac.uk/, accessed on 7 June 2023), and FinnGen (https://www.finngen.fi/en, accessed on 7 June 2023). We selected only subjects of European ancestry for inclusion in the analysis (GD: 1678 patients and 456,942 control participants; HT: 15,654 patients and 379,986 control participants). The described methods of assessment and diagnostic criteria for GD and HT are shown in Supplementary Method S1.

All relevant GWAS data were approved by the respective institutional ethics review boards, and no additional ethical review was required for the published data of this MR study.

### 2.2. Mendelian Randomization

Before delving into the results, we aligned the exposure and outcome data by deducing the forward strand alleles utilizing allele frequency data and excluding palindromic genetic variants (kb > 10,000 and r^2^ < 0.001) [52]. Additionally, we assessed the potency of the genetic instruments for all SNPs using the F-statistic derived as (β^2^/se^2^), followed by an MR analysis focusing on IVs with an F-statistic surpassing 10 [53].

Our primary analytical approach centered around employing the inverse-variance weighting (IVW) method [54]. IVW calculates the weighted average of each IV’s effect estimate, using the square of its standard error as the weight, to estimate the overall effect of exposure on the outcome. We used Cochran’s Q test for heterogeneity, and when heterogeneity existed, we used the multiplicative random-effects model of IVW [54]. When *p* < 0.05 for the IVW method, we used the weighted median [55] and Mendelian randomization pleiotropy residual sum and outlier (MRPRESSO) [56] regression for sensitivity analysis to strengthen its robustness. The weighted median approach, based on the median effect of the IVs rather than the mean, reduces bias in the presence of pleiotropic IVs. MRPRESSO offers an advanced method for detecting and correcting pleiotropy, performing a global test and identifying outliers among the IVs; outliers are then adjusted or removed to improve estimation accuracy. To gauge pleiotropy, we employed the intercept term of the MR–Egger regression and the MRPRESSO global test [56]. In addition, we performed “leave-one-out (LOO)” analysis [56], sequentially excluding each IV to verify result stability. To exclude IVs more closely associated with the outcome than with the exposure, we executed an additional Steiger test [57], which compares the explanatory power of the instruments for exposure versus the outcome. If an instrument explains more of the exposure than the outcome, this supports the assumed causal direction. More detailed information on these MR methods is provided in Supplementary Method S2.

Vujkovic-Cvijin et al. identified BMI and the frequency of alcohol consumption as influential factors affecting the composition of the host gut microbiota [58]. Therefore, we performed multivariate MR (MVMR) analyses. Furthermore, Cochran’s Q test and the MR-Egger intercept were employed to examine study heterogeneity and detect horizontal pleiotropy, respectively. In instances where heterogeneity arose within the IVW method, we employed the weighted median approach to assess the impact of BMI and alcohol consumption frequency on causal inference. Detailed information regarding the origins and characteristics of the GWAS data for BMI and alcohol consumption frequency can be found in Supplementary Table S3.

### 2.3. Statistical Analysis

We performed a total of (116 + 9) × 3 = 375 primary IVWs. To reduce the risk of false positives, we applied the FDR (using the Benjamini–Hochberg method) multiple-test correction (using a 5% false discovery rate). We considered a significant causal association between exposure and outcome when *p* < 0.05 and FDR < 0.05 and a suggestive association when *p* < 0.05 but FDR ≥ 0.05 [59]. We conducted the data analysis using R (version 4.1.2) and applied the R packages “TwoSampleMR” [57] (version 0.5.6), “MendelianRandomization” (version 0.9.0) [60], and “MRPRESSO” [56] (version 1.0).

## 3. Results

The objective of this study was to test the hypothesis that the gut microbiota and its associated metabolites play a causal role in AITD. Participants were exclusively from European populations, and there was no significant overlap between the samples used for exposures and outcomes. We only included exposures with more than three independent SNPs (r^2^ < 0.01 and *p* < 1 × 10^−5^) and an F-statistic > 10. All pooled datasets that were used to obtain study exposures and outcomes are presented in Supplementary Tables S4 and S6.

### 3.1. Impact of Gut Microbiota Genus Abundance on AITD, GD, and HT

**Figure 2:**
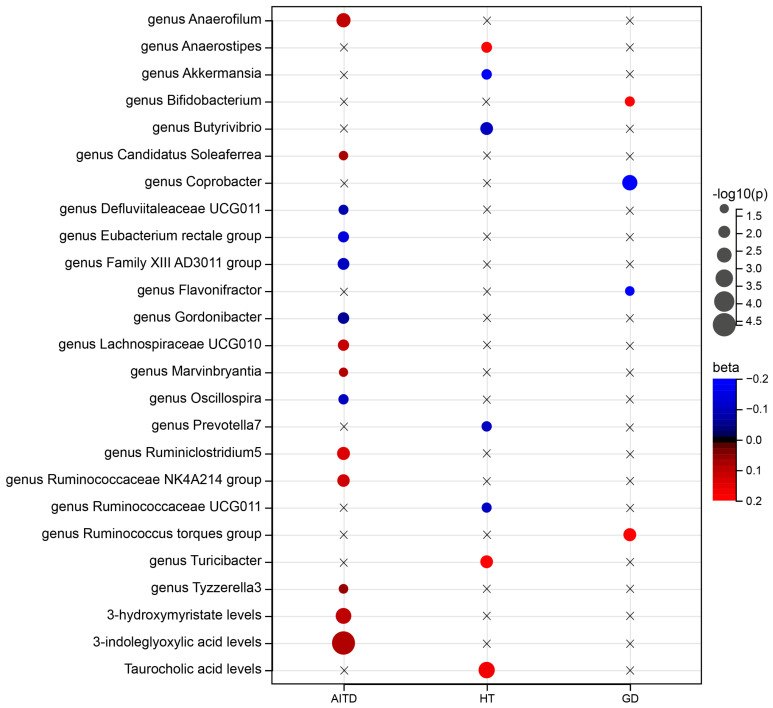
Balloon plot of association of gut microbiota and associated metabolites with three AITD outcomes. Significance of associations of 25 exposures with one or more outcomes is depicted (IVW: *p* < 0.05). Associations at *p* > 0.05 are depicted with crosses. Beta means magnitude of potential causal effect of gut microbiota traits or metabolites on thyroid disease. Beta > 0 indicates that changes in gut microbiota may increase risk of thyroid disease, while beta < 0 suggests that gut microbiota may have protective effect on thyroid health.

Out of the 348 primary IVW analyses, only 22 passed a significance test with a threshold of *p* < 0.05 (Figure 2 and Supplementary Table S5). An increase in the abundance of seven genera (*Anaerofilum*, *Candidatus Soleaferrea*, *Lachnospiraceae UCG0101*, *Marvinbryantia*, *Ruminiclostridium5*, *Ruminococcaceae NK4A214* group, and *Tyzzerella3*) showed potential causal associations with AITD, while five genera (*Defluviitaleaceae UCG011*, *Eubacterium rectale* group, *Family XIII AD3011* group, *Gordonibacter*, and *Oscillospira*) exhibited potential protective effects against AITD. Two genera, *Bifidobacterium* and *Ruminococcus torques* group, were found to have potential pathogenic effects on GD, while *Coprobacter* and *Flavonifractor* had potential protective effects against GD. *Anaerostipes* and *Turicibacter* had potential causal effects on HT, and *Akkermansia*, *Butyrivibrio*, *Prevotella7*, and *Ruminococcaceae UCG011* had potential protective effects against HT. However, these associations no longer existed after FDR (Benjamini–Hochberg) correction (Supplementary Table S5).

### 3.2. Impact of Gut Microbiota Metabolites on AITD, GD, and HT

Out of the 24 primary analyses conducted, only three had statistically significant results (*p* < 0.05) (Figure 2 and Supplementary Table S7). The levels of 3-hydroxymyristate and 3-indoleglyoxylic acid were identified as potential causative factors for AITD, while taurocholic acid levels were identified as potential causative factors for HT (Figure 2 and Supplementary Table S7). After correcting for FDR, only a significant positive causal relationship between levels of circulating 3-indoleglyoxylic acid and AITD remained. The risk of AITD increased by 9% for each standard deviation increase in 3-indoleglyoxylic acid levels (OR = 1.09, 95% CI = 1.05 to 1.14, *p* = 2.43 × 10^−5^, FDR = 0.009) (Figure 2 and Supplementary Table S7).

### 3.3. Sensitivity Analysis, Pleiotropy Test, and MVMR Analysis

**Figure 3:**
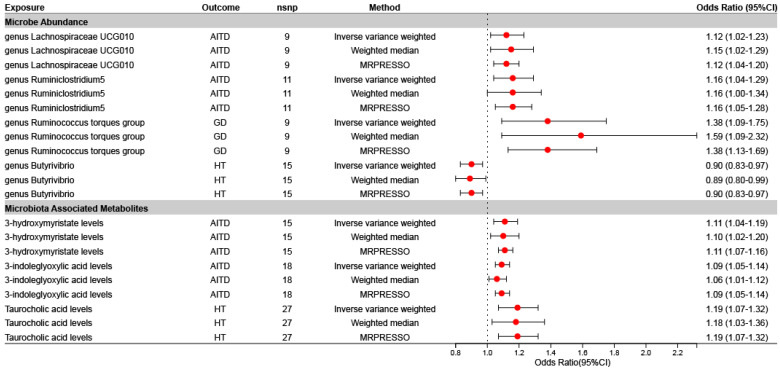
The forest plots illustrate the relationship between the seven exposures and outcomes with robust agreement across robust MR analysis methods. Dots depict the odds ratios (OR). Horizontal bars depict the 95% confidence intervals (CIs). The OR is used to quantify the potential causal effect of specific exposures on the risk of outcomes. OR > 1 indicates that the gut microbiota and associated metabolite characteristics are associated with an increased risk of thyroid disease; OR < 1 suggests that the gut microbiota and associated metabolite characteristics may have a protective effect against thyroid disease. AITD, autoimmune thyroid disease; GD, Graves’ disease; HT, Hashimoto’s thyroiditis; MRPRESSO, Mendelian randomization pleiotropy residual sum and outlier.

To check the robustness of the results, we performed sensitivity analyses for the above 25 exposures and outcomes with potential causality (IVW: *p* < 0.05). Weighted median and MRPRESSO regressions were used, with each test making different assumptions regarding potential pleiotropy. The estimates from the different tests were consistent, supporting the existence of robust causality. Pleiotropy was also assessed using the MR-Egger intercept and the MRPRESSO Global test. Seven associations were retained after excluding nonrobust results (weighted median *p* > 0.05, MRPRESSO *p* > 0.05, MR-Egger intercept *p* < 0.05, MRPRESSO Global test < 0.05) (Figure 3 and Supplementary Table S8), and all IVs were subjected to a Steiger filter test, which showed that the IVs used in the analysis did not have reverse causal relationships with the outcomes (Supplementary Tables S4 and S6). Our analysis revealed a strong positive correlation between circulating 3-indoleglyoxylic acid levels and AITD, which was consistent across multiple analytical approaches. The weighted median approach yielded an OR of 1.06 (95% CI: 1.01 to 1.12, *p* = 0.025), while the MRPRESSO approach yielded an OR of 1.09 (95% CI: 1.05 to 1.14, *p* = 0.001). Additionally, LOO analysis confirmed the robustness of all SNPs (Supplementary Figure S1).

**Figure 4:**
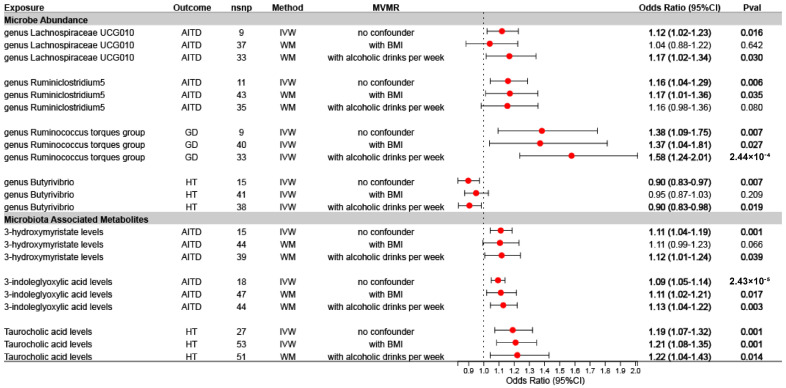
The MVMR results before and after correcting for BMI and alcohol consumption frequency. Dots depict the odds ratio (OR). Horizontal bars depict the 95% confidence intervals (CIs). The OR is used to quantify the potential causal effect of specific exposures on the risk of outcomes. OR > 1 indicates that the gut microbiota and associated metabolite characteristics are associated with an increased risk of thyroid disease; OR < 1 suggests that the gut microbiota and associated metabolite characteristics may have a protective effect against thyroid disease. No confounders, no adjustments were made; with BMI, MVMR adjusted for BMI as a confounding factor; with alcoholic drinks per week, MVMR adjusted for the frequency of alcohol consumption. AITD, autoimmune thyroid disease; BMI, body mass index; GD, Graves’ disease; HT, Hashimoto’s thyroiditis; IVW, inverse-variance weighting; MVMR, multivariate mendelian randomization; WM, weighted median. Bold, *p* < 0.05.

Further MVMR analysis with BMI and frequency of alcohol consumption as confounders showed that circulating 3-indoleglyoxylic acid levels increased the risk of AITD and were not confounded by BMI and alcohol consumption (with BMI: OR = 1.11, 95% CI = 1.02 to 1.21, *p* = 0.017; with alcoholic drinks per week: OR = 1.13, 95% CI = 1.04–1.22, *p* = 0.003) (Figure 4). Similarly, the potential association of the *Ruminococcus torque* group with GD (with BMI: OR = 1.37, 95% CI = 1.04 to 1.81, *p* = 0.027; with alcoholic drinks per week: OR = 1.58, 95% CI = 1.24 to 2.01, *p* = 2.44 × 10^−4^), and the potential causal role of circulating taurocholic acid level on HT (with BMI: OR = 1.21, 95% CI = 1.08 to 1.35, *p* = 0.001; with alcoholic drinks per week: OR = 1.22, 95% CI = 1.04 to 1.43, *p* = 0.014) were also unaffected by BMI and frequency of alcohol consumption, which may warrant further investigation.

## 4. Discussion

We performed a comprehensive two-sample MR analysis of 119 gut microbiota exposures and nine metabolite exposures on three AITD outcomes in order to estimate the causal relationship. We found that genetically determined elevated levels of 3-indoleglyoxylic acid were associated with an increased risk of AITD in a European population and were not affected by BMI and frequency of alcohol consumption. In addition, the ability of the *Ruminococcus torques* group to potentially increase the risk of GD, and the potential pathogenic role of circulating taurocholic acid level on HT, were also not affected by BMI and frequency of alcohol consumption.

The strong familial and heritable nature of AITD development prompts attention to important susceptibility genes common to the development of AITD [61,62]. The abnormal expression of immune-related genes resulting from the polymorphism of some thyroid-related immune regulatory genes can lead to the collapse of the immune system and the development of disease [61]. Some immune-related susceptible genes, such as FOXP3, CTLA-4, PTPN22/LYP, FCRL3, etc., have been found in both GD and HT [61]. These genes affect thyroid autoimmunity at various stages [61]. The partial consistency of genetic susceptibility and the similarity in the pathogenesis of GD and HT allow some genetic epidemiological studies to integrate the genetic information of AITD together and conduct subsequent analyses [6,63]. Our MR analysis is based on previously reported integrated GWAS data and large blood and fecal histology studies.

The influence of the gut microbiota, a novel environmental factor, on thyroid disease appears to be less direct due to biological distance limitations. Based on the gut–thyroid axis hypothesis, the role of gut flora-related metabolites in the circulation is particularly important [8]. Tryptophan derivatives are an important gut microbiota metabolite for immune regulation [64]. Tryptophan can be catabolized by aromatic amino acid aminotransferases (ArAT) into various indole derivatives [65]. These indole derivatives are known ligands for the immunoregulatory transcription factor aryl hydrocarbon receptor (AhR), which interacts with transcription factors such as NF-κB, estrogen receptor, etc. [66], and is capable of activating regulatory feedback loops highly relevant to immunoregulation [67]. 3-indoleglyoxylic acid is a derivative of indole-3-acetic acid [42,68]. A recent study on microbiology revealed that 3-indoleglyoxylic acid enhances autoimmunity and promotes IL-17 production by T cells in vitro [42]. Furthermore, there may be a causal relationship between 3-indoleglyoxylic acid and multiple sclerosis [42]. In our study, through MR analysis, we found that circulating 3-indoleglyoxylic acid in plasma showed a significant pathogenic role in AITD and was not disturbed by BMI or frequency of alcohol consumption. The role played by 3-indoleglyoxylic acid in AITD and even in autoimmune diseases should be explored in depth.

In addition to metabolites, we also evaluated a wide range of gut microbiota genera. In the primary IVW analysis, there may have been 22 genera potentially associated with AITD; however, after correction for multiple testing, all of these associations disappeared. Although the *Ruminococcus torques* group was able to potentially increase the risk of GD, in the LOO analysis, the potential causal association of *Ruminococcus torques* group with GD disappeared after excluding rs10904297 (Supplementary Figure S2). This suggests that the potential causal association of *Ruminococcus torques* group with GD was driven by a single SNP (rs10904297); therefore, robust conclusions cannot yet be drawn. Molecular simulations have shown that certain *Bifidobacterium* and *Lactobacillus* strains may induce AITD through a cross-reactive antigen-mimetic mechanism due to their amino acid sequence homology with human thyroid peroxidase and thyroglobulin [69]. In our study, the IVW method revealed that the same *Bifidobacterium* nominally increased the risk of GD, which somewhat supports the conclusions of molecular modeling. However, in our study, no genetically determined increase in *Lactobacillus* abundance was found to be associated with any type of AITD risk. In addition, it has been shown that some gut microbiota, such as *Yersinia pestis* and *Helicobacter pylori*, produce antigenic cross-reactive substances with the thyroid-stimulating hormone receptor, which affects thyroid autoimmunity [70]. Unfortunately, the 119 genera analyzed in this study did not include these pathogenic bacteria, and their causal relationship with AITD could not be further verified. The many discrepant genera previously reported in observational studies may be influenced by many confounding factors, such as diet, race, alcohol consumption, and BMI, which are difficult to control for. The MVMR analyses we performed can compensate for this lack of control for confounding factors. In addition, we selected participants with European ancestry only to meet the requirements of MR analysis and to avoid the influence of mixed race on the relationship between gut microbiota and disease. In conclusion, invalid results accounted for a large proportion of our MR analyses of gut microbiota and AITD. There may be some publication bias in the large number of positive results presented in recently published research articles [71] and the causal relationship between flora and AITD may be overestimated if not analyzed with correction for multiple testing. It is also possible that altered gut flora abundance is a consequence rather than a cause of AITD. The ability to explore the direction of causality is precisely the outstanding advantage of MR analysis for problem solving.

Our article also has some limitations. First, although our MR analysis included 119 genera and 9 metabolites, some genera and metabolites were still not included. Second, the threshold for SNP selection in our analysis did not reach the strict threshold level (*p* < 5 × 10^−8^), but instead used the threshold commonly used in gut microbiota and metabolite research (*p* < 1 × 10^−5^). However, we removed all weak IVs. Third, in addition to BMI and alcohol consumption, environmental factors and even seasons have important effects on gut flora, but we have not yet been able to exclude the influence of these factors by analytical means. In future clinical studies on the gut microbiota, dietary intake can be assessed through food records or questionnaires, while detailed lifestyle factors (e.g., physical activity, sleep quality, and stress levels) are evaluated and adjusted for in the analysis. Consistency in the season of microbiota sampling should also be maintained. Additionally, longitudinal studies can investigate seasonal fluctuations in the gut microbiota, and statistical methods may be used to minimize seasonal impact on microbiota composition. Fourth, we only examined the gut microbiota at the genus level, as this was the smallest classification identified in the original GWASs. Since higher levels, such as phylum or family, are more likely to include both increasing and decreasing genera, a mixed result would be expected. Further technical and data improvements, such as identification to the species or even subspecies level, are needed for future studies. Finally, although we selected the European population for our study to minimize bias due to ethnicity, this also limits the extrapolation of our results.

Although there are limitations in the IV assumptions in MR analysis, and there may be residual confounding or other alternative causal pathways, this study provides a good reference for future investigations between the gut microbiota and AITD in clinical practice. In addition, our study shows, for the first time, that the level of metabolites associated with the gut microbiota may be a causative factor for AITD to some extent. Intervention strategies targeting specific gut bacteria, such as tryptophan-metabolizing bacteria and *Ruminococcus torques*, may hold promise for targeted biotherapy in AITD management. Future research could further elucidate the gut–thyroid axis by exploring temporal changes in the gut microbiota of AITD patients and examining how the key microbial composition and associated metabolite levels evolve over the course of the disease. Additionally, future work could focus on analyzing the impact of other environmental factors, such as diet and lifestyle, on the gut microbiota to address potential confounders and strengthen causal conclusions. These approaches can meaningfully expand the translational impact of gut microbiota research on the management of AITD.

## 5. Conclusions

In conclusion, our MR analysis suggests a possible pathogenic effect of circulating 3-indoleglyoxylic acid on AITD, highlighting the impact of tryptophan derivatives on AITD. Additionally, the *Ruminococcus torques* group may increase GD risk, and elevated taurocholic acid levels may play a pathogenic role in HT. Further research is needed to confirm these findings.

## Data Availability

The datasets supporting the conclusions of this article are available on the GWAS catalog website (https://www.ebi.ac.uk/gwas/, accessed on 7 June 2023), and in the MiBioGen consortium (https://mibiogen.gcc.rug.nl/, accessed on 7 June 2023), UK Biobank (https://www.ukbiobank.ac.uk/, accessed on 7 June 2023), BioBank Japan (https://biobankjp.org/en/, accessed on 7 June 2023), and FinnGen (https://www.finngen.fi/en, accessed on 7 June 2023). The references for these GWAS meta-analyses are shown in Supplementary Table S3.

## Supplementary Materials

The following supporting information can be downloaded at: https://www.mdpi.com/article/10.3390/nu16223898/s1, Method S1: Methods of assessment and diagnostic criteria for diseases; Method S2: Detailed description of Mendelian randomization methods; Figure S1: Plots of leave-one-out analysis for MR estimates of causal effect of 3-indoleglyoxylic acid on AITD; Figure S2: Plots of leave-one-out analysis for MR estimates of causal effect of *Ruminococcus torques* group on GD; Table S1: STROBE-MR checklist of recommended items to address in reports of Mendelian randomization studies; Table S2: Description of nine gut microbiota-associated metabolite selection rationale; Table S3: Details of data sources included in mendelian randomization analysis; Table S4: Harmonized datasets for each gut microbiota abundance trait exposure and outcome; Table S5: Results of IVW analysis and sensitivity analysis; Table S6: Harmonized datasets for each metabolite trait exposure and outcome; Table S7: Results of IVW analysis and sensitivity analysis; Table S8: Results of robust MR analysis; Table S9: Results of MVMR analysis and sensitivity analysis. References [71,72,73] are cited in Supplementary Materials.
