# Supplementary material for: The Gut Microbiota and Its Metabolites and Their Association with the Risk of Autoimmune Thyroid Disease: A Mendelian Randomization Study
Source: Nutrients. 2024 Nov 15;16(22):3898. doi: 10.3390/nu16223898 (PMC11597551; doi:10.3390/nu16223898)
Supplement: Supplementary file 1 [file nutrients-16-03898-s001.zip › Supplementary Figures.pdf]

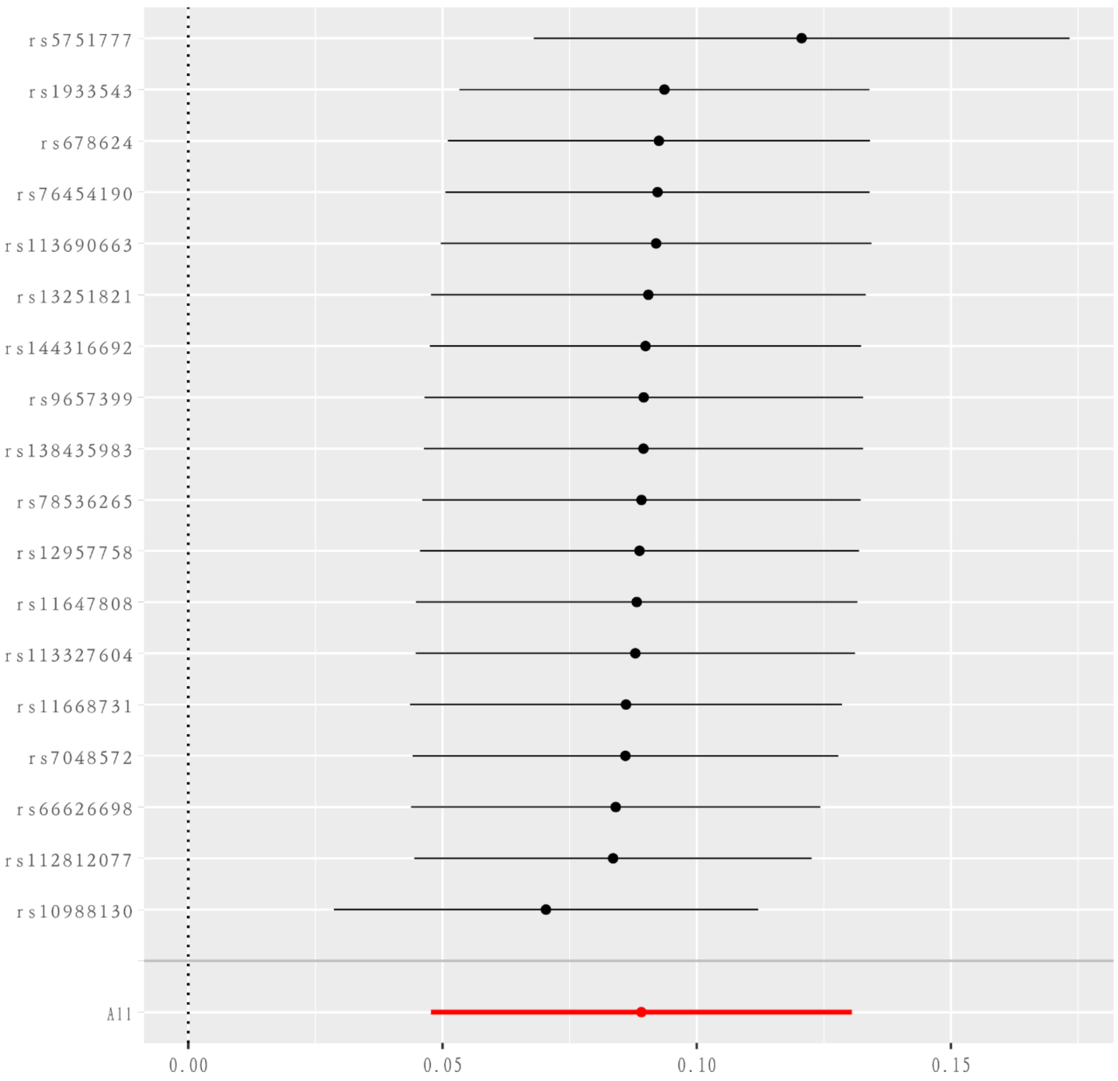

Supplementary Figure S1: Plots of leave-one-out analysis for MR estimates of the causal effect of 3-indoleglyoxylic acid on AITD.

AITD, autoimmune thyroid disease; MR, Mendelian randomization.

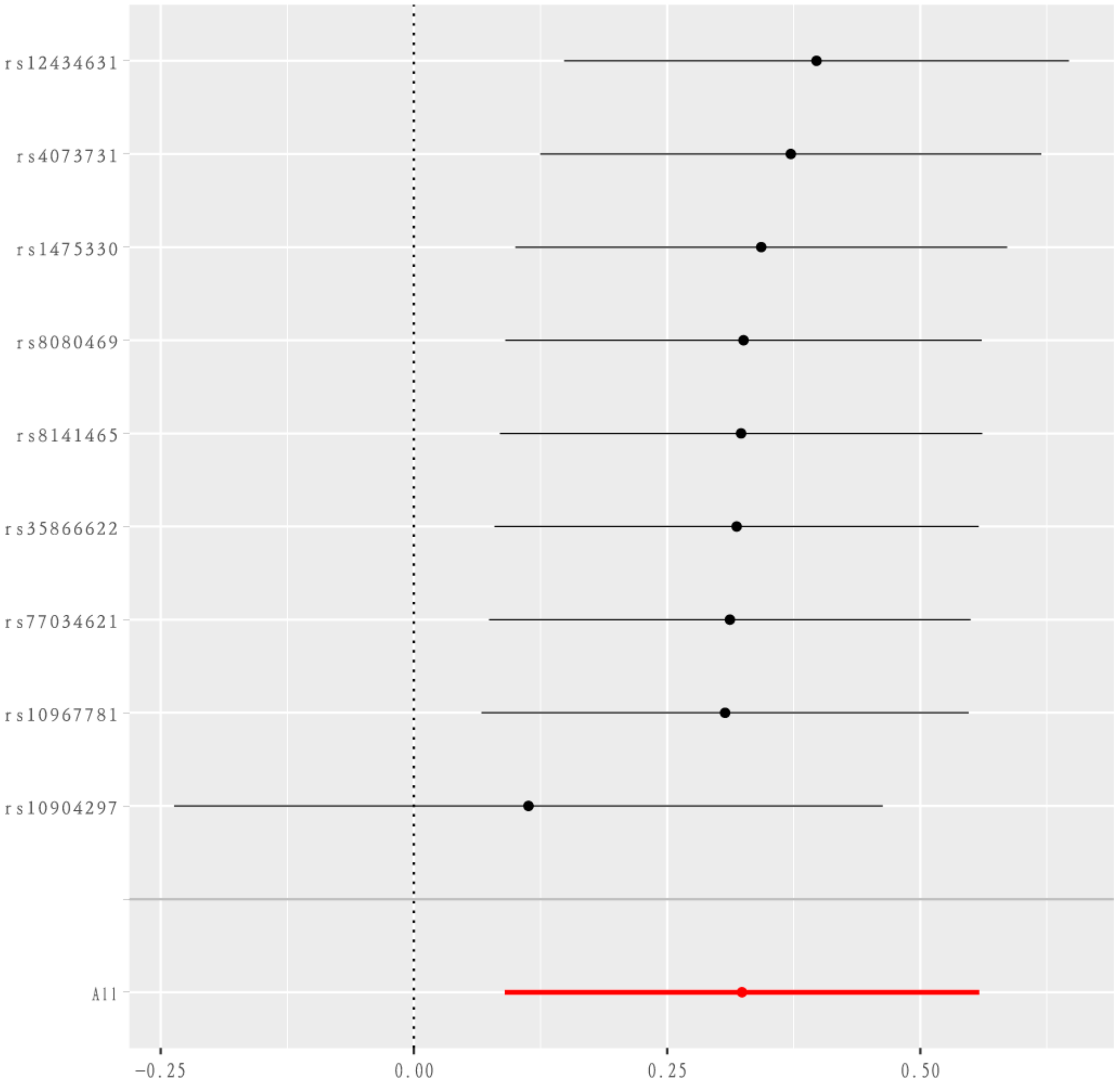

Supplementary Figure S2: Plots of leave-one-out analysis for MR estimates of the causal effect of Ruminococcus torques group on GD.

GD, Graves’ disease; MR, Mendelian randomization.
