# Supplementary material for: The Gut Microbiota and Its Metabolites and Their Association with the Risk of Autoimmune Thyroid Disease: A Mendelian Randomization Study
Source: Nutrients. 2024 Nov 15;16(22):3898. doi: 10.3390/nu16223898 (PMC11597551; doi:10.3390/nu16223898)
Supplement: Supplementary file 1 [file nutrients-16-03898-s001.zip › SupplementaryMethodS1.pdf]

Supplementary Method S1: Methods of assessment and diagnostic criteria for diseases

Traits	Literatures (PMID)	Year	Author	Diagnostic criteria	Sample size	Country
GD	21841780	2011	Chu et al.	Diagnosis of Graves' disease was based on documented clinical and biochemical evidence of hyperthyroidism, diffuse goiter and the presence of at least one of the following: positive TRAb tests, diffusely increased.	Cases:5530<br>Controls:5026	Chinese
GD	21900946	2011	Nakabayashi et al.	Diagnosis of thyroid disease was established based on clinical findings and results of routine examinations; circulating thyroid hormone and thyroid stimulating hormone (TSH) concentrations, serum levels of antibodies against thyroglobulin, thyroid microsomes and TSH receptors, ultrasonography, [99m]TCO4– (or [123I]) uptake and thyroid scintigraphy.	Cases:1551<br>Controls:3875	Japan
GD	22922229	2012	Cooper et al.	GD was defined by the presence of biochemical hyperthyroidism with: 1) a diffuse goiter on a radionuclide or sonographic scan; 2) Graves' ophthalmopathy [no	Cases:2285<br>Controls:9364	European

				signs or symptoms; only signs, no symptoms; signs only; proptosis; eye muscle involvement; corneal involvement; sight visual acuity reduction (NOSPECS) classification score ≥ 2] (22); 3) positive autoantibodies to the TSH receptor; 4) diffuse goiter on physical examination and positive antibodies to thyroglobulin or thyroid peroxidase; or 5) confirmation of a lymphocytic infiltrate in thyroid histology.		
GD	23612905	2013	Zhao et al.	Diagnosis of GD was based on documented clinical and biochemical evidence of hyperthyroidism, diffuse goiter and the presence of at least one of the following: positive TSH receptor antibody tests, diffusely increased 131I (iodine-131) uptake in the thyroid gland or exophthalmos.	Cases:9529<br>Controls:9984	Chinese
GD	25429627	2015	Oryoji et al.	GD patients were diagnosed based on the presence of biochemical hyperthyroidism together with the presence of: 1) a diffuse goiter on a scan; 2) positive autoantibodies to	Cases:547<br>Controls:1363	Japan

				TSHR; or 3) thyroid eye disease.		
GD	26151496	2015	Chen et al.	GD was diagnosed on the basis of the presence of clinical and biochemical hyperthyroidism together with either the presence of thyroid eye disease or a diffuse goitre and a significant titre of autoantibodies	N=1208	Taiwan
GD	27304844	2016	Khong et al.	Graves' disease was defined by the presence of hyperthyroidism based on thyroid function test, including T3, T4, and TSH, and either elevated thyrotropin receptor (TSHR) antibodies or diffuse uptake on technetium-99m pertechnetate thyroid scan.	N=1363	澳大利亚
GD	30067105	2018	Chang et al.	The diagnosis of GD was based on the presence of clinical and biochemical hyperthyr oidism, together with either the presence of thyroid eye dis ease or a diffuse goiter and a significant titer of autoantibodies	Cases:115<br>Controls:40	Chinese
GD	31050781	2019	Zhao et al.	Diagnosis with GD was based on documented clinical and biochemical evidence of hyperthyroidism, diffuse goiter, and the presence of at least one	Cases:2056<br>Controls:3249	Chinese

				of the following clinical characters: positive thyroid-stimulating hormone receptor antibody (TRAb) tests, exophthalmos, or pretibial myxedema.		
GD	32514122	2020	Ishigaki et al.	NA	Cases:2176<br>Controls:210277	Japan
HT	22922229	2012	Cooper et al.	HT was defined by the presence of biochemical hypothyroidism and positive antibodies to thyroglobulin or thyroid peroxidase, the presence of lymphocytic infiltrate in a fine-needle aspirate, or the presence of a diffuse goiter on physical examination.	Cases:462<br>Controls:9364	European
HT	25429627	2015	Oryoji et al.	HT patients were diagnosed based on the presence of biochemical hypothyroidism and diffuse swelling of the thyroid gland together with the presence of: 1) positivity for thyroid peroxidase autoantibodies; 2) thyroglobulin autoantibodies; or 3) lymphocytic infiltration in the thyroid gland	Cases:446<br>Controls:1363	Japan
HT	30284222	2019	Brčić et al.	Diagnosis of HT followed ETA recommendations and guidelines for	Cases:708<br>Controls:735	European

				Management of Subclinical Hypothyroidism.		
HT	30926877	2019	Brčić et al.	Diagnosis was established primarily on the basis of clinical examination, thyroid hormone values, positivity to thyroid antibodies and characteristic thyroid ultrasound (US) image (unhomogenic and/or hypoechogenic thyroid tissue).	N=430	European
HT	32019955	2020	Brčić et al.	Patients were diagnosed with HT on the basis of clinical examination and diffuse thyroid autoimmune disease established by characteristic thyroid ultrasound (US) imaging (unhomogenic thyroid tissue with diffusely reduced echo levels). Diagnosis was further complemented with biochemical measurements of thyroid hormones and antibodies (increased TSH, decreased T3, T4 and/or increased TPOAb and/or TgAb) according to ETA recommendations and guidelines for Management of Subclinical Hypothyroidism	N=345	European

Supplementary Method S2: Detailed description of the Mendelian randomization methods.

Inverse variance weighted (IVW) regression first calculated a Wald estimate for each SNP (i.e., the β coefficient of the exposure SNP divided by the β coefficient of the outcome SNP) and used a combination of meta-analysis methods to obtain an overall estimate of the effect of exposure on the outcome(1). IVW does not take into account the presence of the intercept term and is fitted with the inverse of the ending variance (quadratic of standard error) as the weight. If horizontal pleiotropy is not present or is balanced, a reliable causal estimate can be obtained by IVW analysis(2). When there was heterogeneity, we used the random effects model of IVW.

Weighted median method provides consistent estimates of effect when at least half of the weighted variance provided by horizontal pleiotropy is valid. Compared to the MRPRESSO outlier test, the weighted median method has less bias in the causal estimation smaller but also less precision(3).

MR–Egger regression is assumed to be based on instrumental Strength Independent of Direct Effect (InSIDE), which gives a valid test of the null causal hypothesis and could provide a test for unbalanced pleiotropy and considerable heterogeneity, whereas it requires a larger sample size for the same and may be strongly influenced by outlier genetic variants resulting in inaccurate results. When there is pleiotropy, we prefer the results calculated by the MR–Egger method(4).

MRPRESSO consists of three parts: (a) detection of horizontal pleiotropy (MR-PRESSO global test), (b) correction for horizontal pleiotropy by outlier removal (MR-PRESSO outlier test), and (c) test for significant differences in causal estimates before and after outlier correction (MRPRESSO distortion test)(3). It is a method for identifying and correcting outliers in IVW regression. The MRPRESSO outlier test requires that valid IVs be at least 50%, have balanced pleiotropy, and meet the InSIDE requirement that instrument exposure and pleiotropy effects are irrelevant. When the percentage of horizontal pleiotropy is small (≤10%), the MR-PRESSO outlier-adjusted causal estimates have better precision than MR–Egger. However, the opposite is true

when the percentage of horizontal pleiotropy is high (≥50%)(3). These methods make the assessment results more robust.

1. Burgess S, Dudbridge F, Thompson SG. Combining information on multiple instrumental variables in Mendelian randomization: comparison of allele score and summarized data methods. Statistics in medicine. 2016;35(11):1880-906.
2. Hemani G, Zheng J, Elsworth B, Wade KH, Haberland V, Baird D, et al. The MR-Base platform supports systematic causal inference across the human phenome. Elife. 2018;7.
3. Verbanck M, Chen CY, Neale B, Do R. Detection of widespread horizontal pleiotropy in causal relationships inferred from Mendelian randomization between complex traits and diseases. Nature genetics. 2018;50(5):693-8.
4. Burgess S, Thompson SG. Interpreting findings from Mendelian randomization using the MR-Egger method. Eur J Epidemiol. 2017;32(5):377-89.
